# Cognitive network reconstruction in individuals who use opioids compared to those who do not: Topological analysis of cognitive function through graph model and centrality measures

**DOI:** 10.3389/fpsyt.2022.999199

**Published:** 2023-01-04

**Authors:** Elnaz Gharahi, Shiva Soraya, Hamidreza Ahmadkhaniha, Bahman Sadeghi, Mandana Haghshenas, Ali Bozorgmehr

**Affiliations:** ^1^Department of Psychiatry, School of Medicine, Research Center for Addiction and Risky Behavior (ReCARB), Iran University of Medical Sciences (IUMS), Tehran, Iran; ^2^Department of Biochemistry, Institute of Biochemistry and Biophysics (IBB), University of Tehran, Tehran, Iran

**Keywords:** opioid use disorder, graph model, cognitive network, betweenness centrality, closeness centrality, divided attention

## Abstract

**Introduction:**

Cognitive dysfunction related to opioid use disorder (OUD) requires investigation of the interconnected network of cognitive domains through behavioral experiments and graph data modeling.

**Methods:**

We conducted n-back, selective and divided attention, and Wisconsin card sorting tests and reconstructed the interactive cognitive network of subscales or domains for individuals who use opioids and controls to identify the most central cognitive functions and their connections using graph model analysis. Each two subscales with significant correlations were connected by an edge that incorporated in formation of interactive networks. Each network was analyzed topologically based on the betweenness and closeness centrality measures.

**Results:**

Results from the network reconstructed for individuals who use opioids show that in the divided attention module, reaction time and number of commission errors were the most central subscales of cognitive function. Whereas in controls, the number of correct responses and commission errors were the most central cognitive measure. We found that the subscale measures of divided attention module are significantly correlated with those of other tests. These findings corroborate that persons who use opioids show impaired divided attention as higher reaction time and errors in performing tasks. Divided attention is the most central cognitive function in both OUD subjects and controls, although differences were observed between the two groups in various subscales.

**Discussion:**

Although equal proportions of males and females may be used in future studies, divided attention and its subscales may be the most promising target for cognitive therapies, treatments and rehabilitation as their improvement can enhance overall cognitive domain performance.

## 1. Introduction

Previous reports show that the number of individuals who use drugs has increased about 33% from 1990 to 2017, reaching about 7.7 million people worldwide. The latest report published by the United Nations Office on Drugs and Crime (UNODC) in 2021 reveals that around 275 million people use drugs worldwide, while over 36 million people suffered from drug use disorders (1). This increase has occurred mostly in the regions with low, low-middle and middle socio-demographic index. Meanwhile, opioid use disorder (OUD) accounted for most of the cases with its proportion increasing from 47.18% in 1990 to 53.1% in 2017 (2), and continues to account for the largest burden of disease attributed to drug use (1).

OUD is characterized as a chronic relapsing disorder and a successful recovery could be difficult or almost impossible as the individual who use opioids may have the tendency to relapse. In general, the possibility of OUD following the first opioid use is high compared with most other drugs (3). A complex interplay of genetic, developmental, behavioral, and social risk factors likely plays a role in the development of OUD. Evidences indicate that men, individuals with lower levels of education and lower income, non-natives, individuals who are divorced, patients who suffer from chronic pain, and patients with psychiatric disorders such as depression and mania are more likely to become individuals who use opioids (4).

Generally, the class of opioids includes opium and heroin which are extracted directly from the poppy plant (*Papaver somniferum*), synthetic opioids such as fentanyl, and pain relievers available legally by prescription such as oxycodone, hydrocodone, codeine, methadone, and morphine (5). All opioids have the same core structure and affect the same receptors in the brain and body. The most common side effects of opioid use are drowsiness, confusion, nausea, constipation, euphoria, and slowed breathing (6). However, in case of overdose it can also lead to death. According to the World Health Organization (WHO), more than 70% of about 500,000 drug-associated deaths are related to opioids (1).

In addition to opioid agonists such as buprenorphine, methadone and naltrexone which are the most common medications used to treat opioid use disorder (7), non-pharmacological treatments such as exercise therapy, cognitive behavioral therapy (CBT), group support activities, mindfulness, stress reduction, patient education and more recently cognitive rehabilitation have also been administered (8–10).

A large body of evidence demonstrates that people who use opioids suffer from increased motor impulsivity, impaired strategic planning (11), substantial deficits in working memory (12), attention (13), cognitive flexibility and speed of mental processing (14), problem-solving skills (15) and increased risky decision-making (16). However, it is still unclear that which cognitive domain is most affected by opioid use, or which cognitive domain should be prioritized if a cognitive rehabilitation approach is used. Also, not enough evidence is available to justify enhancement of other cognitive domains in individuals who use opioids if only a single specific cognitive domain shows improvement.

In this study, we first evaluated and compared the basic cognitive functions of a group of individuals who use opioids and a group who did not use opioids as the control group. Then, correlation analysis has been conducted between each two estimated subscales of different cognitive tests and showed every significant correlation between each two subscales by a link shown as edges of an interactive network that we reconstructed for each group. These networks were analyzed topologically and the most important nodes of each network were identified by betweenness and closeness centrality values. This approach which is mainly based on graph theory in mathematics has previously been employed to analyze interactive gene networks in studies on different psychiatric disorders (17, 18) to identify the nodes such as genes or test subscales that play the most important role in controlling the flow of information in that network. In fact, the topology of the entire network can be notably modified through adjusting the activity of these most central nodes. Therefore, by identifying the most prominent cognitive domains that might be susceptible or impaired in individuals who use opioids compared to those who do not, it is possible to understand the differences in cognitive networks between the two groups and to target the most important cognitive function in individuals who use opioids in order to potentially improve their whole cognitive profile.

## 2. Materials and methods

### 2.1. Subjects

In this study, 53 subjects including 50 males and 3 females were assigned into two groups of control (*n* = 20) and individuals who use opioids (*n* = 33), and were selected based on the beta power of 95% and alpha of 0.05. The age of the participants was between 18 and 60 years old, and their level of education was between primary school and master’s degree (Supplementary Table 1). All subjects were screened for neurological conditions or medical history, and none of them had major psychiatric comorbidities or acute physical disabilities, and any intellectual disability was also considered as exclusion criteria. All procedures performed were in accordance with the ethical criteria of the APA, institutional and/or national research committee and with the 1964 Helsinki declaration and its later amendments or comparable ethical standards. All participants entered the study with informed consent, and the study was approved by the Ethics Committee of the Iran University of Medical Sciences, No. IR.IUMS.FMD.REC.1400.108.

The individuals who use opioids were all selected based on the diagnosis of two experienced psychiatrists and according to the criteria in the Diagnostic and Statistical Manual of Mental disorders (DSM-V). All individuals who were using opioids were Persian native speakers and all had a history of opioid substance use such as smoking opium and heroin for at least 6 months. The group that use opioids was not in treatment and did not receive any treatments while admitted. They were hospitalized for 1-7 days when they were examined. Also, following the examination of our two psychiatrists, participants and controls were not diagnosed for any major mental disorders or psychiatric comorbidities, and none of them had obvious physical disabilities.

After recording demographic information such as age, gender, level of education and marital status, all participants underwent cognitive assessment using the n-back test, the WCST, and the SDA test. All persons who used opioids were in a steady state without any withdrawal signs when they completed the tasks.

### 2.2. The n-back test

The n-back task is one of the most classical and well-established cognitive paradigms for studying working memory (WM). WM is defined as a cognitive system of limited-capacity that provides temporary storage space as well as required information for cognitive functions such as learning, reasoning and language comprehension (19). The n-back task was originally introduced by Kirchner as a visuo-spatial test of four load factors (“0-back” to “3-back”) (20), and then by Mackworth as a visual letter task with up to six load factors that present letters or pictures as stimulus sequences (21). Basically, n-back task engages multiple processes, such as selection, decision making, suppression and interference separation (22). For each item in the sequence, the participant must decide whether the current stimulus matches the one displayed “n” trials ago (23). Thus, the subject not only needs the storage and continuous information updating in WM, but also requires interference resolution (24). In fact, the participant requires to monitor a series of stimuli and then to respond once the stimulus (i.e., letters, numbers or pictures) is similar to that of the previous n trials, where n is a pre-specified integer as 1, 2, or 3. Responses such as wrong response, no response and correct response as well as the reaction time (ms) were selected as four subscales of n-back task in this study.

### 2.3. The Wisconsin card sorting test

Developed by David Grant and Esta Berg, the WCST is a neuropsychological test that is mainly used to measure executive function and higher-level cognitive abilities such as perseverance, abstract thinking, cognitive flexibility, and set shifting (25, 26). The WCST consists of four stimulus cards and 64 response cards and there are various geometric shapes in different dimensions (colors, forms and numbers). The participants are expected to identify the specific sorting rule and accurately match every response card with one of four stimulus cards through the feedback based on a rule (25). The sorting rule that the participants identify through a process of trial and error is the dimension that each card should be correctly matched. For instance, a response card with three red stars can be matched according to color (red), form (star), or number (three). Following each response, the subject receives feedback (i.e., “correct” or “incorrect”) that is employed to establish the correct sorting rule. Normally, the sorting rule changes without previous warning after ten correct responses in a row which is referred as completing a category and the subject should start the task again to establish the new sorting rule for the next category. Different subscales that were studied in this task include number of categories completed, perseverative errors, other types of errors, correct or wrong responses, number of total tries and tries to complete the first level, total time (s), conceptual level responses, and failure to continue on a specific pattern. The WCST ends when either all of the six categories are completed or 128 trials are done.

### 2.4. The selective and divided attention test

Here, the SDA test which is a continuous performance task (CPT), is divided into two modules. In the selective attention module, participants need to press a special key on the keyboard at maximum speed if they see a predetermined item, and to restrain their response if they see other stimuli (27). In the divided attention module, participants are requested to press specific keys if they see one or both of the predetermined items in their prespecified locations, and if they see other stimuli or if they see the predetermined items in the location other than their prespecified locations, they need to restrain their response (28).

### 2.5. Statistical analysis and cognitive network reconstruction

The statistical indices of centrality and dispersion of the distribution, including mean and standard deviation were used to describe the demographic characteristics as well as the performance results of each group in the cognitive tests. In order to compare the nominal demographic variables between the two groups, the Fisher’s exact test was applied.

The multivariate generalized linear model (MGLM) was used to compare the dependent variables between the two groups. This model provides a regression analysis and analysis of variance for multiple dependent variables by one or more factor variables or covariates.

In order to find possible functional interactions between subscales, the correlations between each two subscales were calculated by the non-parametric Spearman test in the two groups (Supplementary Tables 2, 3). To reconstruct the cognitive interactive network, the correlation tables were loaded in the Cytoscape, version 3.8.2. Cytoscape is an open source platform for visualizing interactive cognitive networks and biological pathways in an integrative manner (29). Functional interactive networks were reconstructed in such a way that each node represents one of the evaluated subscales and each edge represents a statistically significant correlation.

### 2.6. Topological analysis of the reconstructed network

In order to analyze the networks topologically and to determine the size of each node, the betweenness centrality was calculated for each node, using “igraph” package in R ver. 3.4.0 (30). Betweenness centrality, *C*_*B*_, is an invariant of graph that indicates the number of times a node acts as a bridge along the shortest path between two other nodes. In other words, for a vertex *v* ∈ *V*(*G*):


$$C_{B}\,\left(v\right)=\sum_{s\neq v\neq t\in V\,\left(G\right)}\frac{\sigma_{s\,t}\,\left(v\right)}{\sigma_{s\,t}}$$

where σ_*st*_ is the total number of shortest paths from node *s* to node *t* and σ_*st*_(*v*) is the number of those paths that pass through *v* (31). Size of each node in the networks were adjusted to display its centrality magnitude. The larger the size of a node, the more central role it plays in transmitting information between more pairs of nodes. Based on edge betweenness, thickness of edges is adopted to represent the difference in betweenness centralities and to better differentiate the most central values.

Furthermore, closeness centrality (*CC*) is shown as assigned colors to each of the nodes and was used as an index of importance of a vertex within a given complex network that measures how close a vertex is to all other vertices in the graph as an index of average node distance. Nodes whose color shifted to the red spectrum have a higher *CC* value, meaning that they are closer to other nodes in terms of network topography. However, nodes whose color changed to the blue spectrum have a lower *CC* value, meaning that they are farther in relation to other nodes. Thus, the centrality of a node’s closeness indicates how far that node is on average from other associated nodes and in fact, how much they are involved in effectively directing connections in a network. The *CC* is calculated through the following formula:


$$C\,\left(x\right)=\frac{N}{\sum_{y}d\,\left(y,x\right)}$$

where *d*(*y*,*x*)is the distance between nodes *x*and *y*, and *N*is the number of nodes. The node properties including mean *C*_*B*_ (Supplementary Figure 2) and *CC* (Supplementary Figure 3) for each of the subscales are estimated separately for each group. Thickness of each edge was considered proportional to their betweenness. The red spectrum indicates positive correlations between nodes and the blue spectrum shows negative correlations. In order to find the most important links represented as edges, *C*_*B*_of each link was multiplied by its correlation coefficient and thus its weight was calculated (Supplementary Table 5).

## 3. Results

### 3.1. Demographic data analysis

Fisher’s exact test indicated that there is no significant difference between the two groups in terms of demographic variables including education level (*p* = 0.549), female to male ratio (*p* = 0.338), and marital status (*p* = 0.059; Supplementary Table 1). The mean age of the individuals who use opioids and controls was 29.94 ± 9.19 and 36.05 ± 7.75, respectively, and following the U Mann-Whitney test, there was a statistically significant difference between the two groups [*z* = –2.55, *p* = 0.011]. Therefore, age was considered as a covariate in other analyzes.

### 3.2. MGLM analysis of n-back, SDA and WCST scores

**FIGURE 1:**
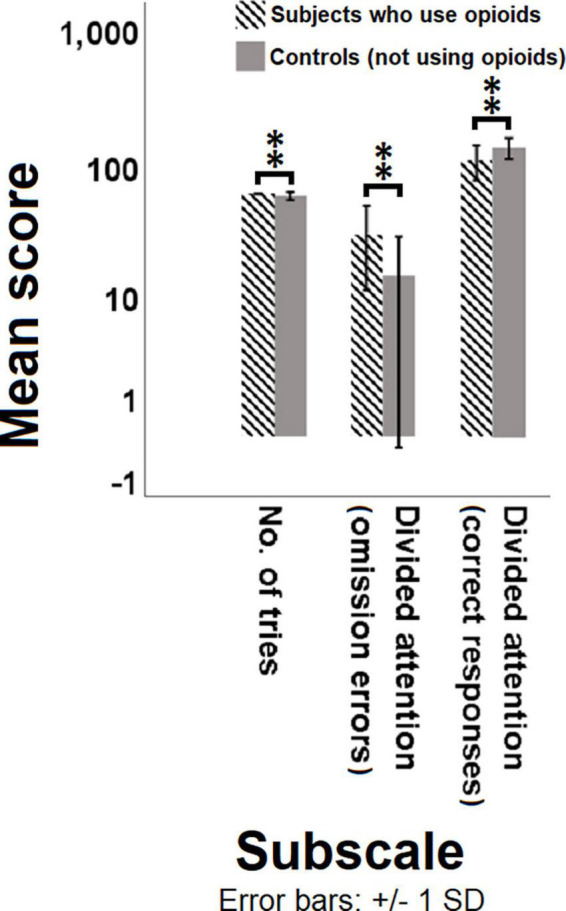
Comparison of the mean score of different test subscales between subjects with opioid use disorder and controls. Multivariate generalized linear model (MGLM) was used to compare the dependent variables of subscales in different tasks between the individuals who use opioids and controls. Significant differences are observed in the number of tries, omission errors and correct responses between the individuals who use opioids and controls. *P* < 0.05 was considered statistically significant. Bars represent mean scores ± SD. ^**^(*P* < 0.01) significant for mean score of subscales between the two groups.

The MGLM analysis showed that the two groups differed significantly in a number of subscales including the number of tries in the WCST [*F*(1, 53) = 7.452, *p*-value = 0.009, $\eta_{p}^{2}$ = 0.0171], and divided attention omission errors [*F*(1, 53) = 11.270, *p*-value = 0.002, $\eta_{p}^{2}$ = 0.184], and divided attention correct responses [*F*(1, 53) = 7.902, *p*-value = 0.007, $\eta_{p}^{2}$ = 0.170] in the SDA test (Figure 1, Supplementary Figure 1, and Supplementary Table 4). Furthermore, remarkably more omission errors in divided attention module during the SDA task show a potentially diminished divided attention for the group that use opioids compared to the control group. This is also corroborated by notably more correct responses in the same task and the same module performed by controls.

### 3.3. Cognitive function analysis through interactive network reconstruction

**FIGURE 2:**
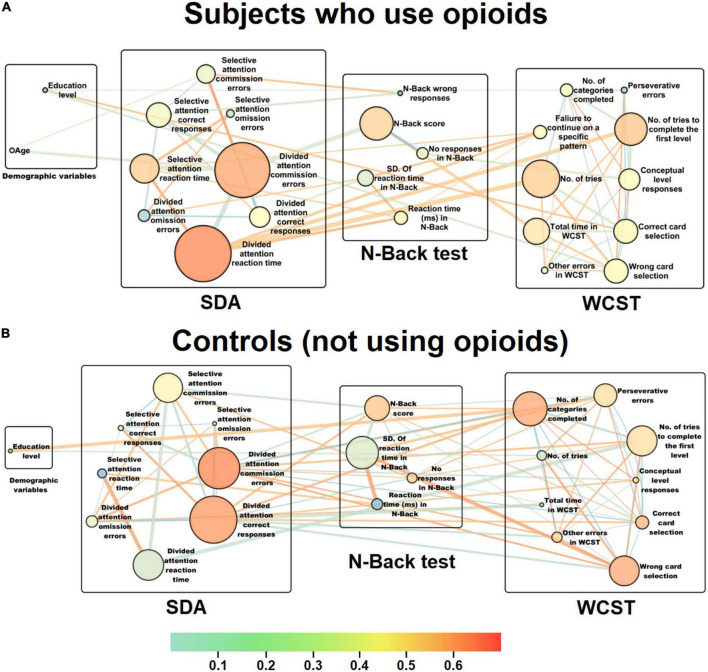
Representation of the interactive cognitive domain networks for the individuals who use opioids and controls. Following cognitive task such as n-back, WCST and SDA, scores for each subscale were collected and after series of correlation and MGLM analyses, cognitive domain networks were reconstructed for **(A)** subjects with opioid use disorder and **(B)** controls. The circular nodes represent the cognitive functions or different subscales within every task. The size of the nodes represents betweenness centrality (*C*_*B*_) and larger nodes mean greater centrality magnitudes. Colors to each of the nodes indicate the closeness centrality (*CC*), as an index of importance of a vertex within a given complex network. This shows how close a vertex is to all other vertices in the graph as an index of average node distance. Nodes whose color shifted to the red spectrum have a higher *CC* value, meaning that they are closer to other nodes in terms of network topography. However, nodes whose color changed to the blue spectrum have a lower *CC* value, meaning that they are farther in relation to other nodes. Thus, the centrality of a node’s closeness indicates how far that node is on average from other associated nodes and in fact, how much they are involved in effectively directing connections in a network. Thickness of each edge was considered proportional to their betweenness. The red spectrum indicates positive correlations between nodes and the blue spectrum shows negative correlations. In order to find the most important links represented as edges, *C*_*B*_ of each link was multiplied by its correlation coefficient and thus its weight was calculated. **(A)** In the reconstructed cognitive network for the subjects with opioid use disorder, the reaction time (*C*_*B*_ = 0.39, *CC* = 0.58) and the number of commission errors (*C*_*B*_ = 0.37, *CC* = 0.56) in the divided attention module of the SDA test show the highest betweenness centrality and closeness centrality, meaning that these two subscales are the most central for the individuals who use opioids. **(B)** In the reconstructed cognitive network for the control subjects, the number of correct responses (*C*_*B*_ = 0.18, *CC* = 0.64) had the highest betweenness centrality, and the number of commission errors (*C*_*B*_ = 0.14, *CC* = 0.65) indicated the highest closeness centrality in the divided attention module of the SDA task, meaning that these two subscales are the most central for the control group.

Following the psychological test score analysis for different task subscales and different cognitive functions, interactive network of those cognitive domains was reconstructed for both groups to illustrate the most central links and cognitive domains with significant contribution to changes in cognitive state of the individuals who use opioids compared to controls. In the reconstructed cognitive network for the subjects with opioid use disorder (Figure 2A), the reaction time (*C*_*B*_ = 0.39, *CC* = 0.58) and the number of commission errors (*C*_*B*_ = 0.37, *CC* = 0.56) in the divided attention module of the SDA test show the highest betweenness centrality (Supplementary Figure 2) and closeness centrality (Supplementary Figure 3), meaning that these two subscales are the most central for the group that use opioids. These data from the centrality measures of the cognitive network support the results from MGLM analysis implying that the persons who use opioids had a significant increase in reaction time or a much lower speed during tasks and more errors indicative of a potential loss of divided attention. Following the calculation of weight of the edges for each of the two linking nodes through multiplication of the *C*_*B*_ measures and correlation coefficients of every two linked subscales, the most important positive link with the largest weight was between the number of tries in the WCST and the reaction time in the divided attention module of the SDA test (43.87). This highly significant positive link between the number of tries and the reaction time justifies the more attempts in performing a certain module of the task in relation to the higher reaction time in SDA and potential loss of attention in the individuals who use opioids. Further, the most important negative link with lowest weight was found to be between the number of commission errors and the reaction time of the divided attention module in SDA test (-58.03). This largely significant negative link between the number of errors and the reaction time also justifies the more errors in performing a certain module of the task in relation to the higher reaction time in SDA and potential loss of attention in the persons who use opioids.

On the other hand, in the reconstructed cognitive network for the control subjects (Figure 2B), the number of correct responses (*C*_*B*_ = 0.18, *CC* = 0.64) had the highest betweenness centrality (Supplementary Figure 2), and the number of commission errors (*C*_*B*_ = 0.14, *CC* = 0.65) indicated the highest closeness centrality (Supplementary Figure 3) in the divided attention module of the SDA task, meaning that these two subscales are the most central for the controls. These results from the centrality measures of the cognitive network also corroborate the data from MGLM analysis implying that the controls had significant correct responses indicative of a better divided attention. Following the estimation of weight of the edges for each of the two linking nodes for every two linked subscales, the most important positive link with the largest weight was between the reaction times of the selective and the divided attention modules in the SDA test (27.27). This largely significant positive link between the reaction times of the attention module justifies the importance of reaction time in both selective and divided modules in maintaining attention during the SDA task. Further, the most important negative link with lowest weight was found to be between the number of tries to complete the first level in the WCST and the reaction time in the divided attention module of the SDA test (-18.43). This highly significant negative link between the number of tries in WCST and the reaction time in SDA also justifies the fewer attempts in performing a certain module of a task such as the first level of the WCST in relation to the lower reaction time in the divided attention module of the SDA test which indicates intact divided attention skills in the control group.

## 4. Discussion

In this study, we first evaluated basic cognitive functions including SDA, working memory and cognitive flexibility; and then compared the subscale scores between subjects with opioid use disorder and the control group. The results showed that the total number of tries in the WCST, and the number of omission errors in the divided attention module in persons who use drugs were significantly higher than the control group. This is while, subjects with opioid use disorder had a notably lower number of correct responses in the divided attention module compared to the control group, implying a remarkable impaired divided attention in individuals who use drugs.

The negative impact of opioid use on cognitive functions has been proven in several studies. For example, Pau et al. found that heroin addiction has a negative effect on impulse control (32). Hekmat et al. reported that subjects with opioid addiction had significantly lower cognitive flexibility, attention and speed of mental processing compared to the controls (33). Furthermore, Yan et al. found that subjects with heroin addiction had remarkably impaired working memory and performed poorly in affective decision-making tasks in comparison with controls (34); and Huili et al. also revealed that switching attention is significantly impaired in individuals who use opioids which may be related to the impairment of their sustained attention function (35). Opioid-induced cognitive impairment can be elaborated through its destructive effect on brain structure and function, as opioids may exert various neurotoxic mechanisms in the brain such as neuronal apoptosis, gray matter loss, mitochondrial and synaptic dysfunctions as well as disruption in neurogenesis (36). Additionally, through using different imaging modalities such as structural magnetic resonance imaging (MRI), diffusion tensor imaging (DTI) and resting-state functional MRI, Upadhyay et al. demonstrated that individuals with opioid use disorder display bilateral volumetric loss in the amygdala and has significantly decreased anisotropy in ventral amygdalo-fugal axonal pathway and uncinate fasciculus as well as the internal and external capsules, and significant decreases in functional connectivity in the anterior insula, nucleus accumbens and amygdala (37).

Subsequently, we reconstructed cognitive interactive networks for both groups based on the correlations between the acquired subscale scores. In the topological network reconstruction for persons who use opioids, the reaction time and the number of commission errors had the highest centrality meaning that the higher reaction time and commission errors are the most important parameters indicative of attention deficits in that particular cognitive domain. Whereas in the control group, the number of correct responses and commission errors in the divided attention module was marked as the most central subscales showing their normal performance within that particular cognitive domain.

Divided attention could be defined as the brain’s ability to attend to two or more different stimuli at the same time, and respond to the multiple demands simultaneously (38). In other words, divided attention is the ability to process different information sources successfully and carry out multiple tasks at a time (38, 39). It is believed that a widespread bilateral network, including dorso- and ventrolateral prefrontal cortex, superior and inferior parietal cortex, and anterior cingulate gyrus are highly involved in divided attention (40). With regards to the divided attention, we found that the most important links shown with larger edge thickness are between the subscales of the divided attention module with the subscales of other tests. In the cognitive network reconstructed for the individuals who use opioids, the most significant positive link was between the number of tries in the WCST and the reaction time in the divided attention module, while the most notable negative correlation was between the number of commission errors and the reaction time of the divided attention module corroborating the significant link between the higher reaction time and errors in response to the WCST task. In the control group network, the most notable positive link was between the reaction time of the selective and the divided attention modules, while the most significant negative correlation was between the number of tries to complete the first level in the WCST and the reaction time in the divided attention module. Significant number of tries in the WCST for the individuals who use opioids compared to controls indicates potential difficulty or dysfunction in higher-level cognitive abilities such as perseverance, abstract thinking, cognitive flexibility, and set shifting. Based on almost all memory models, including the multi-store model of memory (41), the working memory model (42) and the attention to memory model (43), attention is the gateway of information to memory processing. Therefore, poor attention interferes with the flow of information needed for memory processing at higher levels.

From the structural point of view, divided attention requires the coordinated and integrated functioning of different regions of both hemispheres of the brain. As we also indicated, the regions involved in divided attention largely overlap with the regions involved in other cognitive functions. Therefore, dysfunction of the brain regions involved in divided attention can also be associated with dysfunction in other cognitive domains.

Given these two perspectives, and also with respect to the cognitive networks reconstructed in our study, it seems that improving divided attention may moderate the entire cognitive network in both groups of individuals who use opioids and those who do not use drugs as the controls. With follow-up experimental investigations, it is possible to identify the target cognitive functions related to these cognitive domains in different groups of people who use drugs to be able to establish more affordable, time-effective and clinically efficient cognitive therapies in future. In addition, future studies may consider larger and more homogeneous populations, with equal proportions of males and females, and take into account other factors such as dose and duration of drug use. Other cognitive domains, such as planning and problem-solving abilities, risky decision-making, and the level of anxiety or depression can also be assessed to reconstruct far more comprehensive cognitive networks.

## Data availability statement

The original contributions presented in the study are included in the article/Supplementary material, further inquiries can be directed to the corresponding author and BS, bahman.sadeghi@ut.ac.ir; bahmans1983@yahoo.com.

## Ethics statement

The studies involving human participants were reviewed and approved by Ethics Committee of the Iran University of Medical Sciences, No. IR.IUMS.FMD.REC.1400.108. The patients/participants provided their written informed consent to participate in this study.

## Author contributions

EG was involved in subject evaluation and data collection. SS was involved in subject selection and project supervision. HA was involved in subject selection and project supervision, providing supplies and standard monitoring rooms, and standard setups for subject evaluation. BS conducted statistical analysis and manuscript writing, editing, and finalizing. MH provided assistance in subject evaluation and data collection. AB was involved in project design, statistical analysis, and writing the initial manuscript draft. All authors have approved the final manuscript.
