# Supplementary material for: Cognitive network reconstruction in individuals who use opioids compared to those who do not: Topological analysis of cognitive function through graph model and centrality measures
Source: Front Psychiatry. 2023 Jan 4;13:999199. doi: 10.3389/fpsyt.2022.999199 (PMC9846762; doi:10.3389/fpsyt.2022.999199)
Supplement: Supplementary file 1 [file Data_Sheet_1.docx]

Supplementary Material

**Supplementary tables and figures**

|  | | **Group** | | **Statistics** |
| --- | --- | --- | --- | --- |
|  |  | **Individuals who use opioids**  **(n=33)** | **Controls (not using opioids)**  **(n=20)** |  |
| **Education level** | Primary school | 3 | 2 | **Fisher’s exact test:** 4.536 (df=1, *p* =0.549) |
|  | Secondary school | 11 | 8 |  |
|  | High school diploma | 12 | 6 |  |
|  | Associate's degree | 0 | 2 |  |
|  | Master's degree | 7 | 2 |  |
| **Gender** | Male | 32 | 18 | **Fisher’s exact test:** 1.133 (df=4, *p* =0.338) |
|  | Female | 1 | 2 |  |
| **Marital status** | Single | 21 | 6 | **Fisher’s exact test:** 5.651 (df=2, *p* =0.059) |
|  | Married | 11 | 13 |  |
|  | Divorced | 1 | 1 |  |
| **Age** |  | 29.94±9.19 | 36.05±7.75 | **U Mann-Whitney test:** z=-2.55, *p* =0.011 |

**Table S1.** Demographic data representing the statistical analyses for the level of education, gender (female to male ratio), marital status and age in the subjects with opioid use disorder and controls. Fisher’s exact test indicated no significant differences among the two groups in terms of each classification. U Mann-Whitney test indicated a statistically significant difference in age between the two groups [z=-2.55, *p* =0.011]. Therefore, age was considered as a covariate in other analyzes. Age is shown in mean±SD. *p*< 0.05 was considered statistically significant.

| **Domain 1** | **Domain 2** | **Correlation coefficient** | ***p*-value** |
| --- | --- | --- | --- |
| Conceptual level responses | No responses in N-Back | -0.353 | 0.044 |
| Correct card selection | Wrong card selection | -0.974 | 0.001 |
| Correct card selection | Other errors in WCST | -0.62 | 0.001 |
| Correct card selection | Total time in WCST | -0.384 | 0.027 |
| Correct card selection | No. of tries | -0.365 | 0.037 |
| Correct card selection | Education level | 0.454 | 0.008 |
| Correct card selection | Conceptual level responses | 0.729 | 0.001 |
| Divided attention commission errors | Divided attention correct responses | -0.808 | 0.001 |
| Divided attention commission errors | Divided attention reaction time | -0.514 | 0.002 |
| Divided attention commission errors | Age | -0.377 | 0.03 |
| Divided attention omission errors | Divided attention correct responses | -0.748 | 0.001 |
| Faliure to continue on a specific pattern | Selective attention reaction time | 0.412 | 0.017 |
| Faliure to continue on a specific pattern | Divided attention reaction time | 0.414 | 0.017 |
| Faliure to continue on a specific pattern | Reaction time (ms) in N-Back | 0.477 | 0.005 |
| Faliure to continue on a specific pattern | SD. Of reaction time in N-Back | 0.488 | 0.004 |
| N-Back score | Divided attention commission errors | -0.381 | 0.029 |
| N-Back wrong responses | Selective attention correct responses | -0.426 | 0.013 |
| N-Back wrong responses | Selective attention commission errors | 0.503 | 0.003 |
| No responses in N-Back | N-Back score | -0.944 | 0.001 |
| No. of categories completed | Wrong card selection | -0.774 | 0.001 |
| No. of categories completed | Other errors in WCST | -0.714 | 0.001 |
| No. of categories completed | No. of tries | -0.476 | 0.005 |
| No. of categories completed | Perseverative errors | -0.456 | 0.008 |
| No. of categories completed | No. of tries to complete the first level | -0.417 | 0.016 |
| No. of categories completed | Correct card selection | 0.752 | 0.001 |
| No. of categories completed | Conceptual level responses | 0.881 | 0.001 |
| No. of tries | Divided attention reaction time | 0.353 | 0.044 |
| No. of tries | Other errors in WCST | 0.451 | 0.008 |
| No. of tries to complete the first level | Conceptual level responses | -0.485 | 0.004 |
| No. of tries to complete the first level | Divided attention reaction time | 0.366 | 0.036 |
| Other errors in WCST | Conceptual level responses | -0.694 | 0.001 |
| Other errors in WCST | No. of tries to complete the first level | 0.4 | 0.021 |
| Perseverative errors | Correct card selection | -0.79 | 0.001 |
| Perseverative errors | Conceptual level responses | -0.524 | 0.002 |
| Perseverative errors | Education level | -0.518 | 0.002 |
| Perseverative errors | Wrong card selection | 0.754 | 0.001 |
| Reaction time (ms) in N-Back | Selective attention reaction time | 0.433 | 0.012 |
| Reaction time (ms) in N-Back | SD. Of reaction time in N-Back | 0.572 | 0.005 |
| Reaction time (ms) in N-Back | Divided attention reaction time | 0.572 | 0.001 |
| SD. Of reaction time in N-Back | Divided attention correct responses | -0.35 | 0.046 |
| SD. Of reaction time in N-Back | Divided attention omission errors | 0.366 | 0.036 |
| Selective attention commission errors | Selective attention correct responses | -0.817 | 0.001 |
| Selective attention commission errors | Divided attention correct responses | -0.681 | 0.001 |
| Selective attention commission errors | Age | -0.373 | 0.033 |
| Selective attention commission errors | Divided attention commission errors | 0.725 | 0.001 |
| Selective attention correct responses | Divided attention omission errors | -0.535 | 0.001 |
| Selective attention correct responses | Divided attention commission errors | -0.526 | 0.002 |
| Selective attention correct responses | Divided attention correct responses | 0.681 | 0.001 |
| Selective attention omission errors | Divided attention omission errors | 0.537 | 0.001 |
| Selective attention omission errors | Selective attention reaction time | 0.541 | 0.001 |
| Selective attention reaction time | Divided attention commission errors | -0.386 | 0.027 |
| Selective attention reaction time | Divided attention reaction time | 0.615 | 0.001 |
| Total time in WCST | N-Back score | 0.359 | 0.04 |
| Total time in WCST | No. of tries to complete the first level | 0.39 | 0.025 |
| Wrong card selection | Conceptual level responses | -0.743 | 0.001 |
| Wrong card selection | Education level | -0.429 | 0.013 |
| Wrong card selection | Total time in WCST | 0.367 | 0.036 |
| Wrong card selection | No. of tries | 0.404 | 0.02 |
| Wrong card selection | Other errors in WCST | 0.626 | 0.001 |

**Table S2.** Spearmen correlations between each two subscales in subjects with opioid use disorder. In order to find possible functional interactions between subscales, the correlations between each two subscales were calculated by the non-parametric Spearman test. *p*< 0.05 was considered statistically significant.

| **Domain 1** | **Domain 2** | **Correlation coefficient** | ***p*-value** |
| --- | --- | --- | --- |
| Correct card selection | Conceptual level responses | 0.809 | 0.001 |
| No. of categories completed | Conceptual level responses | 0.857 | 0.001 |
| No. of tries to complete the first level | Conceptual level responses | -0.489 | 0.029 |
| Other errors in WCST | Conceptual level responses | -0.824 | 0.001 |
| Perseverative errors | Conceptual level responses | -0.573 | 0.008 |
| Wrong card selection | Conceptual level responses | -0.787 | 0.001 |
| No. of categories completed | Correct card selection | 0.868 | 0.001 |
| Perseverative errors | Correct card selection | 0.886 | 0.001 |
| Perseverative errors | Correct card selection | -0.898 | 0.001 |
| Conceptual level responses | Divided attention commission errors | -0.635 | 0.003 |
| Correct card selection | Divided attention commission errors | -0.719 | 0.001 |
| N-Back score | Divided attention commission errors | -0.52 | 0.019 |
| No responses in N-Back | Divided attention commission errors | 0.579 | 0.007 |
| No. of categories completed | Divided attention commission errors | -0.723 | 0.001 |
| No. of tries to complete the first level | Divided attention commission errors | 0.504 | 0.023 |
| Other errors in WCST | Divided attention commission errors | 0.693 | 0.001 |
| Perseverative errors | Divided attention commission errors | 0.579 | 0.008 |
| Selective attention commission errors | Divided attention commission errors | 0.514 | 0.02 |
| Selective attention correct responses | Divided attention commission errors | -0.577 | 0.008 |
| Selective attention omission errors | Divided attention commission errors | 0.448 | 0.048 |
| Wrong card selection | Divided attention commission errors | 0.715 | 0.001 |
| Conceptual level responses | Divided attention correct responses | 0.462 | 0.04 |
| Correct card selection | Divided attention correct responses | 0.505 | 0.023 |
| Divided attention commission errors | Divided attention correct responses | -0.873 | 0.001 |
| Divided attention omission errors | Divided attention correct responses | -0.902 | 0.001 |
| N-Back score | Divided attention correct responses | 0.519 | 0.019 |
| No responses in N-Back | Divided attention correct responses | -0.569 | 0.009 |
| No. of categories completed | Divided attention correct responses | 0.548 | 0.012 |
| Other errors in WCST | Divided attention correct responses | -0.539 | 0.014 |
| Selective attention commission errors | Divided attention correct responses | -0.59 | 0.006 |
| Selective attention correct responses | Divided attention correct responses | 0.73 | 0.001 |
| Selective attention omission errors | Divided attention correct responses | -0.601 | 0.005 |
| Total time in WCST | Divided attention correct responses | -0.477 | 0.033 |
| Wrong card selection | Divided attention correct responses | -0.523 | 0.018 |
| Divided attention commission errors | Divided attention omission errors | 0.578 | 0.008 |
| Selective attention commission errors | Divided attention omission errors | 0.533 | 0.015 |
| Selective attention correct responses | Divided attention omission errors | -0.712 | 0.001 |
| Selective attention omission errors | Divided attention omission errors | 0.611 | 0.004 |
| Total time in WCST | Divided attention omission errors | 0.554 | 0.011 |
| No. of tries to complete the first level | Divided attention reaction time | -0.475 | 0.034 |
| Selective attention commission errors | Divided attention reaction time | -0.452 | 0.045 |
| Selective attention reaction time | Divided attention reaction time | 0.707 | 0.001 |
| No. of categories completed | Education level | 0.444 | 0.05 |
| No. of tries | Education level | -0.469 | 0.037 |
| Correct card selection | N-Back score | 0.528 | 0.017 |
| No responses in N-Back | N-Back score | -0.969 | 0.001 |
| No. of categories completed | N-Back score | 0.475 | 0.034 |
| Perseverative errors | N-Back score | -0.56 | 0.01 |
| Wrong card selection | N-Back score | -0.505 | 0.023 |
| Correct card selection | No responses in N-Back | -0.546 | 0.013 |
| No. of categories completed | No responses in N-Back | -0.503 | 0.024 |
| No. of tries to complete the first level | No responses in N-Back | 0.447 | 0.048 |
| Perseverative errors | No responses in N-Back | 0.526 | 0.017 |
| Wrong card selection | No responses in N-Back | 0.521 | 0.019 |
| Correct card selection | No. of tries | -0.452 | 0.045 |
| No. of categories completed | No. of tries | -0.696 | 0.001 |
| Perseverative errors | No. of tries | 0.51 | 0.022 |
| Wrong card selection | No. of tries | 0.72 | 0.001 |
| Correct card selection | No. of tries to complete the first level | -0.625 | 0.003 |
| No. of categories completed | No. of tries to complete the first level | -0.586 | 0.007 |
| Other errors in WCST | No. of tries to complete the first level | 0.671 | 0.001 |
| Wrong card selection | No. of tries to complete the first level | 0.602 | 0.005 |
| Correct card selection | Other errors in WCST | -0.788 | 0.001 |
| No. of categories completed | Other errors in WCST | -0.949 | 0.001 |
| No. of tries | Other errors in WCST | 0.769 | 0.001 |
| Perseverative errors | Other errors in WCST | 0.589 | 0.006 |
| Wrong card selection | Other errors in WCST | 0.896 | 0.001 |
| No. of categories completed | Perseverative errors | -0.706 | 0.001 |
| N-Back score | SD. Of reaction time in N-Back | -0.528 | 0.017 |
| Perseverative errors | SD. Of reaction time in N-Back | 0.457 | 0.043 |
| Reaction time (ms) in N-Back | SD. Of reaction time in N-Back | 0.734 | 0.001 |
| Wrong card selection | SD. Of reaction time in N-Back | 0.689 | 0.001 |
| N-Back score | Selective attention commission errors | -0.495 | 0.026 |
| No responses in N-Back | Selective attention commission errors | 0.514 | 0.002 |
| Selective attention commission errors | Selective attention correct responses | -0.662 | 0.001 |
| Selective attention omission errors | Selective attention correct responses | -0.906 | 0.001 |
| Reaction time (ms) in N-Back | Selective attention reaction time | 0.457 | 0.043 |
| Correct card selection | Wrong card selection | -0.944 | 0.001 |
| No. of categories completed | Wrong card selection | -0.931 | 0.001 |

**Table S3.** Spearmen correlations between each two subscales in controls. In order to find possible functional interactions between subscales, the correlations between each two subscales were calculated by the non-parametric Spearman test. *p*< 0.05 was considered statistically significant.

| **Subscale** | **Groups** | **Mean** | **Standard deviation** | **Mean Square** | **F (df=1)** | ***p*-value** | **Partial eta squared ($\eta_{p}^{2}$)** |
| --- | --- | --- | --- | --- | --- | --- | --- |
| **No. of categories completed** | Case | 2.67 | 1.708 | 7.554 | 1.996 | 0.164 | 0.038 |
|  | Control | 3.4 | 2.257 |  |  |  |  |
| **Perseverative errors** | Case | 7.42 | 4.191 | 18.263 | 0.773 | 0.383 | 0.015 |
|  | Control | 6.25 | 5.711 |  |  |  |  |
| **Correct responses** | Case | 32.58 | 7.408 | 47.127 | 0.787 | 0.379 | 0.016 |
|  | Control | 34.35 | 8.093 |  |  |  |  |
| **Wrong responses** | Case | 27.64 | 7.822 | 204.397 | 2.546 | 0.117 | 0.052 |
|  | Control | 23.45 | 10.405 |  |  |  |  |
| **No. of tries** | Case | 59.91 | .384 | 41.898 | 7.452 | 0.009** | 0.0171 |
|  | Control | 57.8 | 3.833 |  |  |  |  |
| **Other errors** | Case | 19.91 | 4.996 | 72.274 | 2.456 | 0.123 | 0.060 |
|  | Control | 17.2 | 5.961 |  |  |  |  |
| **Total time (s)** | Case | 261.85 | 102.087 | 28011.381 | 3.594 | 0.064 | 0.081 |
|  | Control | 224.5 | 62.016 |  |  |  |  |
| **No. of tries to complete the first level** | Case | 18.61 | 16.641 | 0.648 | 0.002 | 0.962 | 0.002 |
|  | Control | 17.85 | 16.503 |  |  |  |  |
| **Conceptual level responses** | Case | 2.91 | 2.887 | 27.655 | 3.505 | 0.067 | 0.090 |
|  | Control | 4 | 2.828 |  |  |  |  |
| **Failure to continue on a specific pattern** | Case | 0.36 | .742 | 0.658 | 1.637 | 0.207 | 0.032 |
|  | Control | 0.15 | .366 |  |  |  |  |
| **Wrong responses** | Case | 9.94 | 9.483 | 77.358 | 0.891 | 0.350 | 0.033 |
|  | Control | 6.8 | 8.847 |  |  |  |  |
| **No response** | Case | 31.94 | 28.673 | 34.014 | 0.037 | 0.848 | 0.002 |
|  | Control | 29.55 | 32.157 |  |  |  |  |
| **Score (Correct responses)** | Case | 78.12 | 27.297 | 213.964 | 0.227 | 0.636 | 0.011 |
|  | Control | 83.65 | 35.040 |  |  |  |  |
| **Reaction time (ms)** | Case | 607.79 | 161.807 | 22229.480 | 0.925 | 0.341 | 0.039 |
|  | Control | 662.95 | 140.561 |  |  |  |  |
| **Selective attention commission errors** | Case | 6.0779 | 1.61807 | 151.316 | 2.481 | 0.122 | 0.180 |
|  | Control | 6.6295 | 1.40561 |  |  |  |  |
| **Selective attention omission errors** | Case | 8.67 | 9.993 | 39.390 | 0.738 | 0.394 | 0.017 |
|  | Control | 3.25 | 2.863 |  |  |  |  |
| **Selective attention correct responses** | Case | 4.42 | 8.295 | 345.113 | 2.453 | 0.124 | 0.096 |
|  | Control | 3 | 5.068 |  |  |  |  |
| **Selective attention reaction time (ms)** | Case | 154.91 | 14.139 | 4162.980 | 0.745 | 0.392 | 0.015 |
|  | Control | 161.75 | 6.488 |  |  |  |  |
| **Divided attention commission errors** | Case | 467 | 83.851 | 505.862 | 1.528 | 0.222 | 0.130 |
|  | Control | 451.15 | 54.119 |  |  |  |  |
| **Divided attention omission errors** | Case | 29.36 | 21.402 | 3412.432 | 11.270 | 0.002** | 0.184 |
|  | Control | 19.2 | 12.722 |  |  |  |  |
| **Divided attention correct responses** | Case | 29.94 | 18.979 | 6671.125 | 7.902 | 0.007** | 0.170 |
|  | Control | 14.2 | 14.406 |  |  |  |  |
| **Divided attention reaction time (ms)** | Case | 108.58 | 31.325 | 5.360 | 0.001 | 0.982 | 0.088 |
|  | Control | 134.6 | 24.106 |  |  |  |  |

**Table S4.** The multivariate generalized linear model (MGLM) was used to compare the dependent variables of subscales in different tasks between the individuals who use opioids and controls. This model provides a regression analysis and analysis of variance for multiple dependent variables by one or more factor variables or covariates. Mean, standard deviation, level of significance (p-value) and partial et-squared ($\eta_{p}^{2}$) as the measure for effect size are shown. *p*< 0.05 was considered statistically significant.


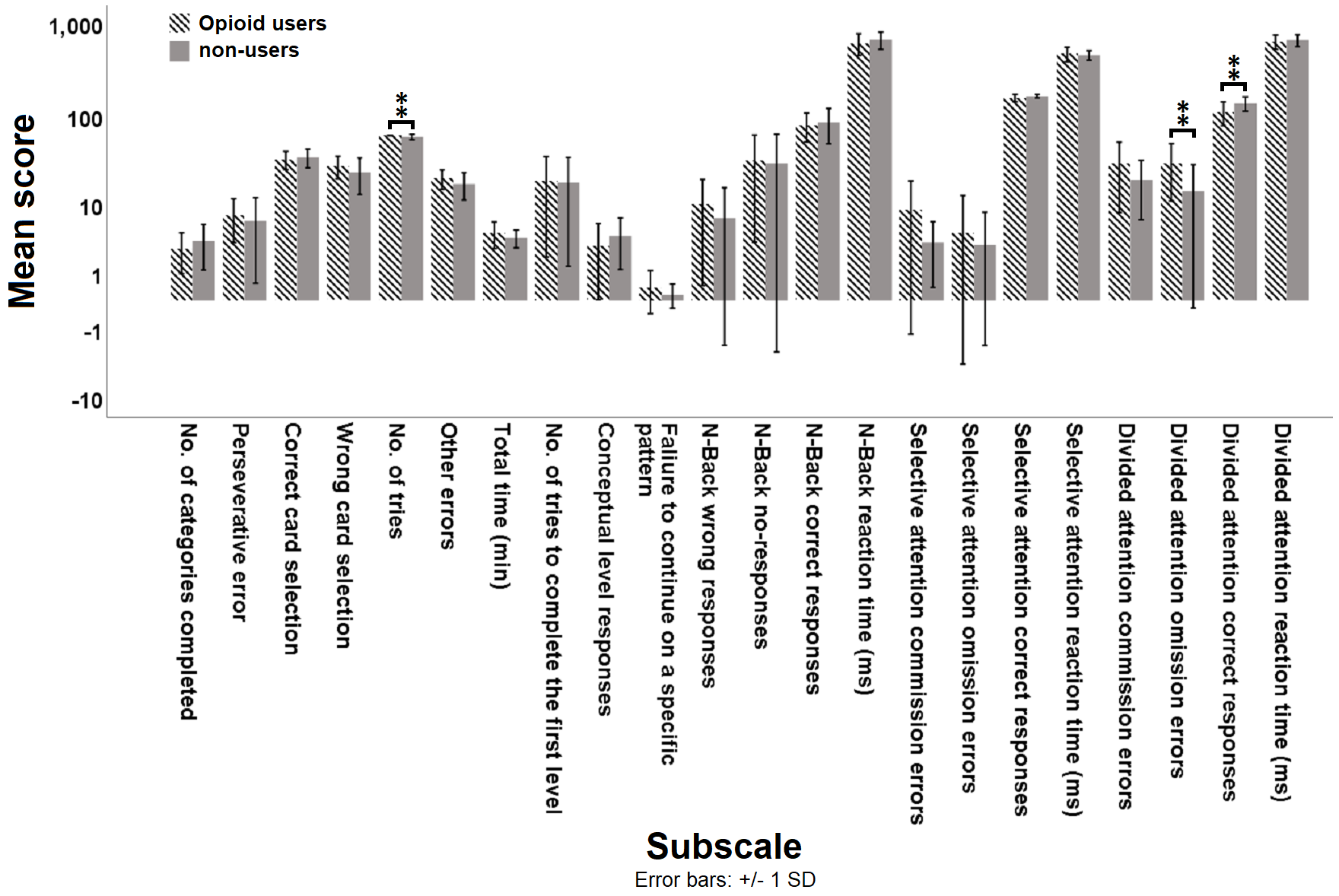


**Supplementary Figure 1.** Comparison of the mean score of different test subscales between subjects with opioid use disorder and controls. Multivariate generalized linear model (MGLM) was used to compare the dependent variables of subscales in different tasks between the individuals who use opioids and controls. Significant differences are observed in the number of tries, omission errors and correct responses between the subjects who use opioids and controls. *P*< 0.05 was considered statistically significant. Bars represent mean scores ± SD. ** (*P*<0.01) significant for mean score of subscales between the two groups.


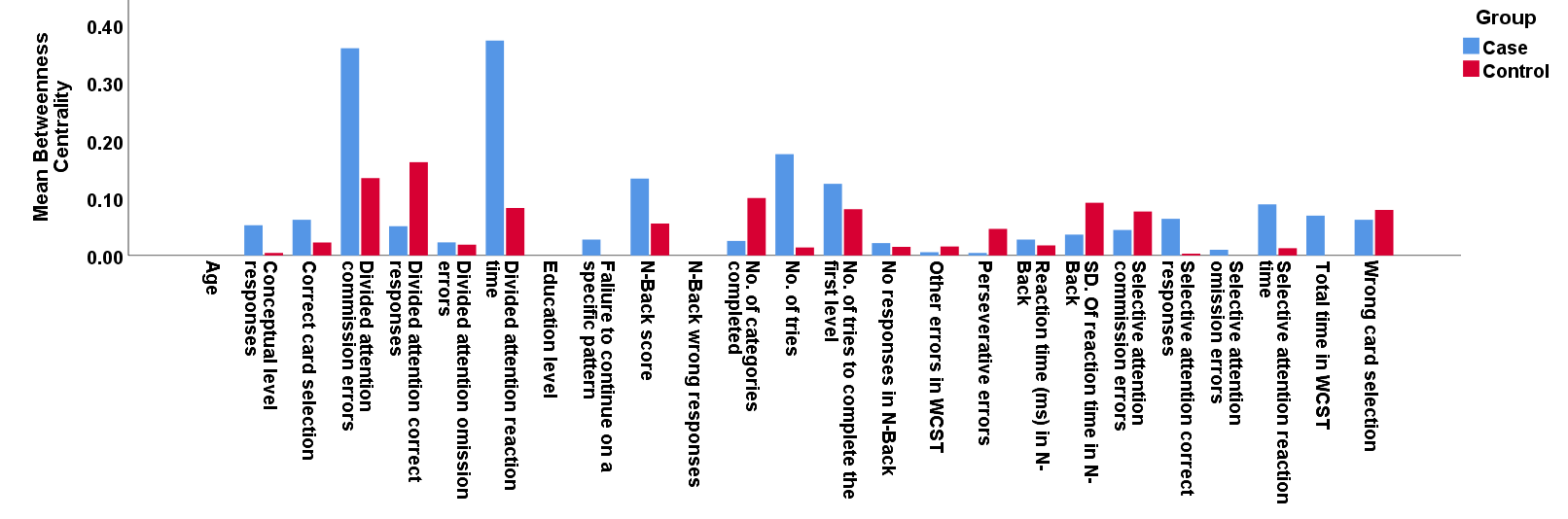


**Supplementary Figure 2.** Comparison of the betweenness centrality (*$C_{B}$*) of different test subscales as cognitive network nodes between the subjects with opioid use disorder and controls.


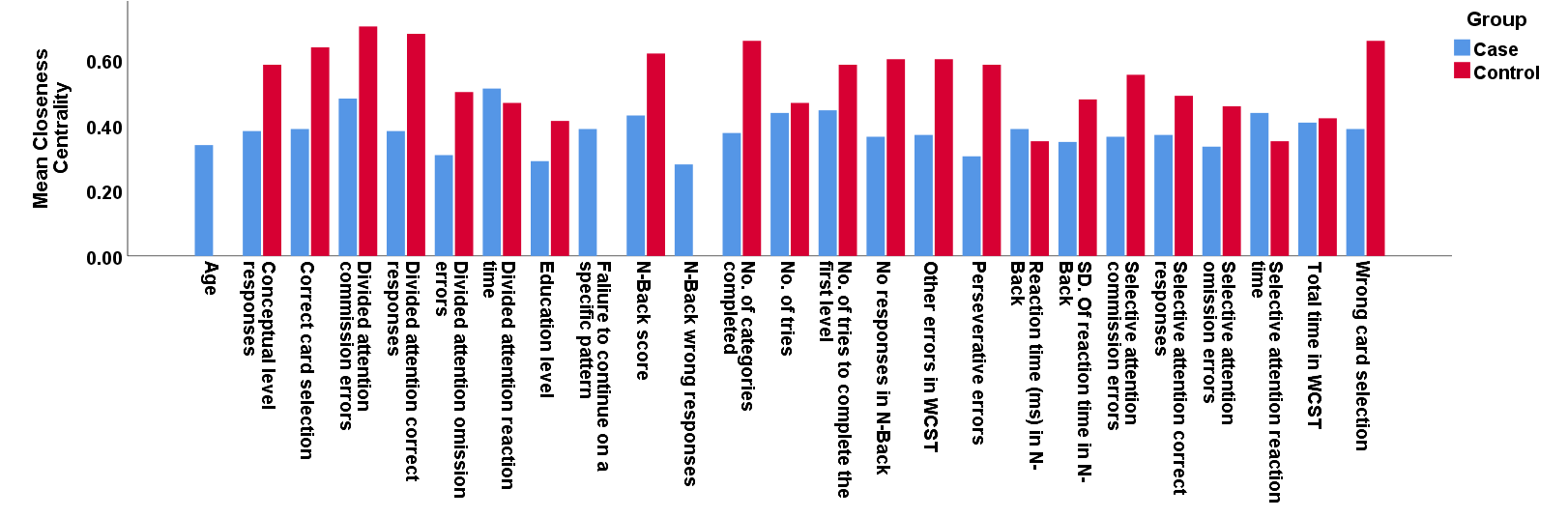


**Supplementary Figure 3.** Comparison of the closeness centrality (*CC*) of different test subscales as cognitive network nodes between the subjects with opioid use disorder and controls.

| **Group** | **Interactions** | **Correlation coefficient** | **Betweenness centrality** | **Weight** | ***p*-value** |
| --- | --- | --- | --- | --- | --- |
| **Case** | No. of tries (interacts with) Divided attention reaction time | 0.353 | 124.2868 | 43.87324 | 0.044 |
| **Case** | Selective attention commission errors (interacts with) Divided attention commission errors | 0.725 | 49.47619 | 35.87024 | 0.001 |
| **Case** | No. of tries to complete the first level (interacts with) Divided attention reaction time | 0.366 | 92.20707 | 33.74779 | 0.036 |
| **Case** | Selective attention reaction time (interacts with) Divided attention reaction time | 0.615 | 45.46544 | 27.96125 | 0.001 |
| **Case** | Reaction time (ms) in N-Back (interacts with) Divided attention reaction time | 0.572 | 42.20353 | 24.14042 | 0.001 |
| **Case** | Selective attention omission errors (interacts with) Selective attention reaction time | 0.541 | 42.1321 | 22.79347 | 0.001 |
| **Case** | Total time in WCST (interacts with) N-Back score | 0.359 | 57.30568 | 20.57274 | 0.04 |
| **Case** | Faliure to continue on a specific pattern (interacts with) Divided attention reaction time | 0.414 | 42.20353 | 17.47226 | 0.017 |
| **Case** | Wrong card selection (interacts with) No. of tries | 0.404 | 39.69877 | 16.0383 | 0.02 |
| **Case** | Reaction time (ms) in N-Back (interacts with) SD. Of reaction time in N-Back | 0.572 | 23.65591 | 13.53118 | 0.005 |
| **Case** | Faliure to continue on a specific pattern (interacts with) SD. Of reaction time in N-Back | 0.488 | 23.65591 | 11.54408 | 0.004 |
| **Case** | N-Back wrong responses (interacts with) Selective attention commission errors | 0.503 | 22.38095 | 11.25762 | 0.003 |
| **Case** | Correct card selection (interacts with) Education level | 0.454 | 21.8 | 9.8972 | 0.008 |
| **Case** | Selective attention omission errors (interacts with) Divided attention omission errors | 0.537 | 16.79877 | 9.020939 | 0.001 |
| **Case** | Wrong card selection (interacts with) Total time in WCST | 0.367 | 24.51951 | 8.99866 | 0.036 |
| **Case** | Perseverative errors (interacts with) Wrong card selection | 0.754 | 10.68495 | 8.056452 | 0.001 |
| **Case** | No. of tries (interacts with) Other errors in WCST | 0.451 | 16.66667 | 7.516668 | 0.008 |
| **Case** | Other errors in WCST (interacts with) No. of tries to complete the first level | 0.4 | 18.23333 | 7.293332 | 0.021 |
| **Case** | Total time in WCST (interacts with) No. of tries to complete the first level | 0.39 | 17.73333 | 6.915999 | 0.025 |
| **Case** | SD. Of reaction time in N-Back (interacts with) Divided attention omission errors | 0.366 | 18.16897 | 6.649843 | 0.036 |
| **Case** | Correct card selection (interacts with) Conceptual level responses | 0.729 | 7.7 | 5.6133 | 0.001 |
| **Case** | No. of categories completed (interacts with) Conceptual level responses | 0.881 | 5.5 | 4.8455 | 0.001 |
| **Case** | Reaction time (ms) in N-Back (interacts with) Selective attention reaction time | 0.433 | 10.40476 | 4.505261 | 0.012 |
| **Case** | Faliure to continue on a specific pattern (interacts with) Selective attention reaction time | 0.412 | 10.40476 | 4.286761 | 0.017 |
| **Case** | Selective attention correct responses (interacts with) Divided attention correct responses | 0.681 | 6.238095 | 4.248143 | 0.001 |
| **Case** | No. of categories completed (interacts with) Correct card selection | 0.752 | 4.533333 | 3.409066 | 0.001 |
| **Case** | Wrong card selection (interacts with) Other errors in WCST | 0.626 | 5.366667 | 3.359534 | 0.001 |
| **Case** | Faliure to continue on a specific pattern (interacts with) Reaction time (ms) in N-Back | 0.477 | 2 | 0.954 | 0.005 |
| **Case** | No. of categories completed (interacts with) Other errors in WCST | -0.714 | 2.5 | -1.785 | 0.001 |
| **Case** | Correct card selection (interacts with) Wrong card selection | -0.974 | 2 | -1.948 | 0.001 |
| **Case** | Perseverative errors (interacts with) Education level | -0.518 | 4.4 | -2.2792 | 0.002 |
| **Case** | Selective attention commission errors (interacts with) Age | -0.373 | 8 | -2.984 | 0.033 |
| **Case** | Correct card selection (interacts with) Other errors in WCST | -0.62 | 5.366667 | -3.32733 | 0.001 |
| **Case** | No. of categories completed (interacts with) Wrong card selection | -0.774 | 4.533333 | -3.5088 | 0.001 |
| **Case** | Other errors in WCST (interacts with) Conceptual level responses | -0.694 | 6 | -4.164 | 0.001 |
| **Case** | Selective attention commission errors (interacts with) Selective attention correct responses | -0.817 | 5.166667 | -4.22117 | 0.001 |
| **Case** | Wrong card selection (interacts with) Conceptual level responses | -0.743 | 7.7 | -5.7211 | 0.001 |
| **Case** | No. of categories completed (interacts with) Perseverative errors | -0.456 | 13.47849 | -6.14619 | 0.008 |
| **Case** | Perseverative errors (interacts with) Conceptual level responses | -0.524 | 13.55161 | -7.10104 | 0.002 |
| **Case** | SD. Of reaction time in N-Back (interacts with) Divided attention correct responses | -0.35 | 22.47619 | -7.86667 | 0.046 |
| **Case** | Selective attention commission errors (interacts with) Divided attention correct responses | -0.681 | 11.7381 | -7.99365 | 0.001 |
| **Case** | Perseverative errors (interacts with) Correct card selection | -0.79 | 10.68495 | -8.44111 | 0.001 |
| **Case** | Wrong card selection (interacts with) Education level | -0.429 | 21.8 | -9.3522 | 0.013 |
| **Case** | Correct card selection (interacts with) Total time in WCST | -0.384 | 24.51951 | -9.41549 | 0.027 |
| **Case** | No. of categories completed (interacts with) No. of tries to complete the first level | -0.417 | 23.65591 | -9.86451 | 0.016 |
| **Case** | No. of categories completed (interacts with) No. of tries | -0.476 | 21.55591 | -10.2606 | 0.005 |
| **Case** | N-Back wrong responses (interacts with) Selective attention correct responses | -0.426 | 25.61905 | -10.9137 | 0.013 |
| **Case** | Conceptual level responses (interacts with) No responses in N-Back | -0.353 | 32.20046 | -11.3668 | 0.044 |
| **Case** | Divided attention omission errors (interacts with) Divided attention correct responses | -0.748 | 15.46851 | -11.5704 | 0.001 |
| **Case** | Selective attention correct responses (interacts with) Divided attention omission errors | -0.535 | 22.70661 | -12.148 | 0.001 |
| **Case** | Selective attention reaction time (interacts with) Divided attention commission errors | -0.386 | 37.47619 | -14.4658 | 0.027 |
| **Case** | Correct card selection (interacts with) No. of tries | -0.365 | 39.69877 | -14.4901 | 0.037 |
| **Case** | Divided attention commission errors (interacts with) Age | -0.377 | 40 | -15.08 | 0.03 |
| **Case** | No. of tries to complete the first level (interacts with) Conceptual level responses | -0.485 | 33.25115 | -16.1268 | 0.004 |
| **Case** | Selective attention correct responses (interacts with) Divided attention commission errors | -0.526 | 58.4447 | -30.7419 | 0.002 |
| **Case** | No responses in N-Back (interacts with) N-Back score | -0.944 | 39.53379 | -37.3199 | 0.001 |
| **Case** | N-Back score (interacts with) Divided attention commission errors | -0.381 | 98.17281 | -37.4038 | 0.029 |
| **Case** | Divided attention commission errors (interacts with) Divided attention correct responses | -0.808 | 47.96851 | -38.7586 | 0.001 |
| **Case** | Divided attention commission errors (interacts with) Divided attention reaction time | -0.514 | 112.9071 | -58.0342 | 0.002 |
| Control | Reaction time (ms) in N-Back (interacts with) SD. Of reaction time in N-Back | 0.734 | 42.7619 | 31.38723 | 0.001 |
| Control | Selective attention reaction time (interacts with) Divided attention reaction time | 0.707 | 38.57143 | 27.27 | 0.001 |
| Control | Wrong card selection (interacts with) SD. Of reaction time in N-Back | 0.689 | 30.23175 | 20.82968 | 0.001 |
| Control | No. of categories completed (interacts with) Education level | 0.444 | 34.4 | 15.2736 | 0.05 |
| Control | Divided attention commission errors (interacts with) Divided attention omission errors | 0.578 | 17.80159 | 10.28932 | 0.008 |
| Control | Perseverative errors (interacts with) SD. Of reaction time in N-Back | 0.457 | 21.52698 | 9.83783 | 0.043 |
| Control | No. of categories completed (interacts with) Divided attention correct responses | 0.548 | 17.85574 | 9.784946 | 0.012 |
| Control | Selective attention correct responses (interacts with) Divided attention correct responses | 0.73 | 12.46032 | 9.096034 | 0.001 |
| Control | Wrong card selection (interacts with) No. of tries | 0.72 | 12.29281 | 8.850823 | 0.001 |
| Control | Selective attention omission errors (interacts with) Divided attention commission errors | 0.448 | 19.61111 | 8.785777 | 0.048 |
| Control | Perseverative errors (interacts with) Divided attention commission errors | 0.579 | 15.00812 | 8.689701 | 0.008 |
| Control | Wrong card selection (interacts with) Divided attention commission errors | 0.715 | 10.27796 | 7.348741 | 0.001 |
| Control | N-Back score (interacts with) Divided attention correct responses | 0.519 | 13.57381 | 7.044807 | 0.019 |
| Control | Selective attention commission errors (interacts with) Divided attention omission errors | 0.533 | 12.5381 | 6.682807 | 0.015 |
| Control | No. of tries to complete the first level (interacts with) Divided attention commission error | 0.504 | 13.20794 | 6.656802 | 0.023 |
| Control | Wrong card selection (interacts with) No. of tries to complete the first level | 0.602 | 10.93571 | 6.583297 | 0.005 |
| Control | Reaction time (ms) in N-Back (interacts with) Selective attention reaction time | 0.457 | 13.90476 | 6.354475 | 0.043 |
| Control | No. of tries (interacts with) Other errors in WCST | 0.769 | 7.701961 | 5.922808 | 0.001 |
| Control | Selective attention commission errors (interacts with) Divided attention commission errors | 0.514 | 11.44379 | 5.882108 | 0.02 |
| Control | Other errors in WCST (interacts with) No. of tries to complete the first level | 0.671 | 8.710317 | 5.844623 | 0.001 |
| Control | Other errors in WCST (interacts with) Divided attention commission errors | 0.693 | 7.803361 | 5.407729 | 0.001 |
| Control | Correct card selection (interacts with) Divided attention correct responses | 0.505 | 10.35574 | 5.229649 | 0.023 |
| Control | No responses in N-Back (interacts with) Selective attention commission errors | 0.514 | 9.359477 | 4.810771 | 0.002 |
| Control | Total time in WCST (interacts with) Divided attention omission errors | 0.554 | 8.5 | 4.709 | 0.011 |
| Control | Perseverative errors (interacts with) No. of tries | 0.51 | 9.175163 | 4.679333 | 0.022 |
| Control | Conceptual level responses (interacts with) Divided attention correct responses | 0.462 | 9.690476 | 4.477 | 0.04 |
| Control | Wrong card selection (interacts with) Other errors in WCST | 0.896 | 4.84127 | 4.337778 | 0.001 |
| Control | No. of categories completed (interacts with) Conceptual level responses | 0.857 | 5.019048 | 4.301324 | 0.001 |
| Control | No. of categories completed (interacts with) N-Back score | 0.475 | 8.817647 | 4.188382 | 0.034 |
| Control | Perseverative errors (interacts with) Correct card selection | 0.886 | 4.646825 | 4.117087 | 0.001 |
| Control | Perseverative errors (interacts with) No responses in N-Back | 0.526 | 6.72479 | 3.53724 | 0.017 |
| Control | Perseverative errors (interacts with) Other errors in WCST | 0.589 | 5.93254 | 3.494266 | 0.006 |
| Control | No. of tries to complete the first level (interacts with) No responses in N-Back | 0.447 | 6.729365 | 3.008026 | 0.048 |
| Control | Correct card selection (interacts with) N-Back score | 0.528 | 5.517647 | 2.913318 | 0.017 |
| Control | No responses in N-Back (interacts with) Divided attention commission errors | 0.579 | 4.904762 | 2.839857 | 0.007 |
| Control | Wrong card selection (interacts with) No responses in N-Back | 0.521 | 5.244631 | 2.732453 | 0.019 |
| Control | Selective attention omission errors (interacts with) Divided attention omission errors | 0.611 | 4.3 | 2.6273 | 0.004 |
| Control | No. of categories completed (interacts with) Correct card selection | 0.868 | 3 | 2.604 | 0.001 |
| Control | Correct card selection (interacts with) Conceptual level responses | 0.809 | 3.019048 | 2.44241 | 0.001 |
| Control | Other errors in WCST (interacts with) Conceptual level responses | -0.824 | 2.4 | -1.9776 | 0.001 |
| Control | Correct card selection (interacts with) No responses in N-Back | -0.546 | 3.689076 | -2.01424 | 0.013 |
| Control | Correct card selection (interacts with) Other errors in WCST | -0.788 | 2.619048 | -2.06381 | 0.001 |
| Control | Selective attention correct responses (interacts with) Divided attention omission errors | -0.712 | 3 | -2.136 | 0.001 |
| Control | Wrong card selection (interacts with) N-Back score | -0.505 | 4.406536 | -2.2253 | 0.023 |
| Control | Perseverative errors (interacts with) N-Back score | -0.56 | 4.931139 | -2.76144 | 0.01 |
| Control | Selective attention omission errors (interacts with) Selective attention correct responses | -0.906 | 3.3 | -2.9898 | 0.001 |
| Control | No. of categories completed (interacts with) No responses in N-Back | -0.503 | 6.189076 | -3.11311 | 0.024 |
| Control | Correct card selection (interacts with) Wrong card selection | -0.944 | 3.555556 | -3.35644 | 0.001 |
| Control | No. of categories completed (interacts with) Other errors in WCST | -0.949 | 3.619048 | -3.43448 | 0.001 |
| Control | Divided attention commission errors (interacts with) Divided attention correct responses | -0.873 | 4.071429 | -3.55436 | 0.001 |
| Control | No. of tries (interacts with) Education level | -0.469 | 7.6 | -3.5644 | 0.037 |
| Control | No. of tries to complete the first level (interacts with) Conceptual level responses | -0.489 | 7.376984 | -3.60735 | 0.029 |
| Control | No responses in N-Back (interacts with) N-Back score | -0.969 | 3.733333 | -3.6176 | 0.001 |
| Control | Perseverative errors (interacts with) Conceptual level responses | -0.573 | 6.33254 | -3.62855 | 0.008 |
| Control | Correct card selection (interacts with) No. of tries | -0.452 | 8.937255 | -4.03964 | 0.045 |
| Control | Wrong card selection (interacts with) Conceptual level responses | -0.787 | 5.24127 | -4.12488 | 0.001 |
| Control | Perseverative errors (interacts with) Correct card selection | -0.898 | 4.646825 | -4.17285 | 0.001 |
| Control | Conceptual level responses (interacts with) Divided attention commission errors | -0.635 | 6.619048 | -4.2031 | 0.003 |
| Control | No responses in N-Back (interacts with) Divided attention correct responses | -0.569 | 7.97619 | -4.53845 | 0.009 |
| Control | No. of categories completed (interacts with) Perseverative errors | -0.706 | 6.446825 | -4.55146 | 0.001 |
| Control | N-Back score (interacts with) Divided attention commission errors | -0.52 | 8.771429 | -4.56114 | 0.019 |
| Control | Correct card selection (interacts with) Divided attention commission errors | -0.719 | 6.684314 | -4.80602 | 0.001 |
| Control | No. of categories completed (interacts with) Wrong card selection | -0.931 | 5.355556 | -4.98602 | 0.001 |
| Control | Correct card selection (interacts with) No. of tries to complete the first level | -0.625 | 8.824603 | -5.51538 | 0.003 |
| Control | No. of categories completed (interacts with) No. of tries | -0.696 | 7.937255 | -5.52433 | 0.001 |
| Control | Other errors in WCST (interacts with) Divided attention correct responses | -0.539 | 11.47479 | -6.18491 | 0.014 |
| Control | Selective attention commission errors (interacts with) Selective attention correct responses | -0.662 | 9.538095 | -6.31422 | 0.001 |
| Control | N-Back score (interacts with) Selective attention commission errors | -0.495 | 14.59757 | -7.2258 | 0.026 |
| Control | Wrong card selection (interacts with) Divided attention correct responses | -0.523 | 15.85812 | -8.2938 | 0.018 |
| Control | No. of categories completed (interacts with) No. of tries to complete the first level | -0.586 | 14.8246 | -8.68722 | 0.007 |
| Control | Selective attention commission errors (interacts with) Divided attention correct responses | -0.59 | 14.77059 | -8.71465 | 0.006 |
| Control | No. of categories completed (interacts with) Divided attention commission errors | -0.723 | 12.18431 | -8.80926 | 0.001 |
| Control | Selective attention omission errors (interacts with) Divided attention correct responses | -0.601 | 14.78889 | -8.88812 | 0.005 |
| Control | Selective attention correct responses (interacts with) Divided attention commission errors | -0.577 | 16.30159 | -9.40602 | 0.008 |
| Control | Divided attention omission errors (interacts with) Divided attention correct responses | -0.902 | 11.46032 | -10.3372 | 0.001 |
| Control | N-Back score (interacts with) SD. Of reaction time in N-Back | -0.528 | 24.11429 | -12.7323 | 0.017 |
| Control | Selective attention commission errors (interacts with) Divided attention reaction time | -0.452 | 33.77143 | -15.2647 | 0.045 |
| Control | Total time in WCST (interacts with) Divided attention correct responses | -0.477 | 33.5 | -15.9795 | 0.033 |
| Control | No. of tries to complete the first level (interacts with) Divided attention reaction time | -0.475 | 38.8 | -18.43 | 0.034 |

**Table S5.** Analytical characteristics of the links for different test subscales in the individuals who use opioids and controls. Mathematical parameters such as betweenness centrality (*$C_{B}$*), interaction effects between each two subscales and their correlation coefficient as well as the level of significance (*p*-value) are provided. Weight of each link in the interactive network reconstruction is calculated by multiplication of *$C_{B}$* and the correlation coefficient. Descending weight measures is set as the table order. *p*< 0.05 was considered statistically significant.
